# Palliative care needs of advanced cancer patients in the emergency department at the end of life: an observational cohort study

**DOI:** 10.1007/s00520-019-04906-x

**Published:** 2019-06-13

**Authors:** Mary-Joanne Verhoef, Ellen de Nijs, Nanda Horeweg, Jaap Fogteloo, Christian Heringhaus, Anouk Jochems, Marta Fiocco, Yvette van der Linden

**Affiliations:** 1 Center of Expertise Palliative Care, Leiden University Medical Center, Albinusdreef 2, 2333 ZA Leiden, the Netherlands; 2 Department of Radiation Oncology, Leiden University Medical Center, Leiden, the Netherlands; 3 Department of Internal Medicine, Leiden University Medical Center, Leiden, the Netherlands; 4 Department of Emergency Medicine, Leiden University Medical Center, Leiden, the Netherlands; 5 Department of Medical Oncology, Leiden University Medical Center, Leiden, the Netherlands; 6 Department of Medical Oncology, Haaglanden Medical Center, The Hague, the Netherlands; 7 Department of Biomedical Data Sciences, Leiden University Medical Center, Leiden, the Netherlands; 8 Mathematical Institute, Leiden University, Leiden, the Netherlands

**Keywords:** Palliative care, Emergency department, Oncology, Terminal care

## Abstract

**Purpose:**

Patients with advanced cancer commonly visit the emergency department (ED) during the last 3 months of life. Identification of these patients and their palliative care needs help initiating appropriate care according to patients’ wishes. Our objective was to provide insight into ED visits of advanced cancer patients at the end of life.

**Methods:**

Adult palliative patients with solid tumours who died < 3 months after their ED visit were included (2011–2014). Patients, ED visits, and follow-up were described. Factors associated with approaching death were assessed using Cox proportional hazards models.

**Results:**

Four hundred twenty patients were included, 54.5% was male, median age 63 years. A total of 54.6% was on systemic anti-cancer treatments and 10.5% received home care ≥ 1 per day. ED visits were initiated by patients and family in 34.0% and 51.9% occurred during out-of-office hours. Dyspnoea (21.0%) or pain (18.6%) were most reported symptoms. Before the ED visit, limitations on life-sustaining treatments were discussed in 33.8%, during or after the ED visit in 70.7%. Median stay at the ED was 3:29 h (range 00:12–18:01 h), and 319 (76.0%) were hospitalized. Median survival was 18 days (IQ range 7–41). One hundred four (24.8%) died within 7 days after the ED visit, of which 71.2% in-hospital. Factors associated with approaching death were lung cancer, neurologic deterioration, dyspnoea, hypercalcemia, and jaundice.

**Conclusion:**

ED visits of advanced cancer patients often lead to hospitalization and in-hospital deaths. Timely recognition of patients with limited life expectancies and urgent palliative care needs, and awareness among ED staff of the potential of ED-initiated palliative care may improve the end-of-life trajectory of these patients.

## Background

Although cancer has become a chronic disease in many patients, still yearly 8.9 million patients die of widespread disease worldwide, which makes cancer a leading cause of death in developed countries [1]. To provide advanced cancer patients with a good quality at the end of life, integration of appropriate palliative care into standard care is essential [2]. Palliative care is driven by patients’ care needs and wishes and must be offered while the illness is not yet life-threatening [2]. One important aspect of a good quality of end of life denoted by patients and their families is to be cared for at home and to die there [3, 4]. Because identification of patients with limited life expectancies and urgent palliative care needs can be difficult, patients, family, and their health care professionals are often not timely prepared and educated about appropriate management of problems expected in the future given the disease trajectory. Advance care planning about patient’s wishes and goals of care often take place too late. Consequently, many patients with advanced cancer and a limited life expectancy are admitted to an emergency department (ED) [5–7], leading to hospital admissions [8] and in-hospital deaths [6, 7]. Commonly reported physical problems in patients with advanced cancer visiting the ED are pain, respiratory distress, gastro-intestinal problems, fatigue, disease progression, delirium and loss of consciousness [5, 8–11]. One study found that the most common reason for ED visits in the last 2 weeks of life was not being able to cope with the situation at home [5]. Other reasons for patients and families to go to the ED are anxiety related to the disease; being defaulted to previously used health care services; feeling safe in and familiar with the hospital setting; and difficulties accessing community health care services, especially when the complaints were urgent or occurred during out-of-office hours [12, 13]. It is plausible that although patients consider ED visits as unwanted and as a ‘last-resort’ solution for relieve of their problems, their distress caused by their disease and care burden leads to these ED visits [13]. ED physicians perceive several barriers to provide appropriate palliative care: the ED is an uncomfortable setting for dying patients [14], physicians work under time pressure which makes palliative patients a low priority [15], they lack confidence in their own palliative care skills [16], do not build a long-lasting relationship with palliative patients and are consequently not comfortable with discussing limitations on medical treatments [15, 17].

Identification of advanced cancer patients with palliative care needs and a short life expectancy at the ED can help to improve the quality of the end of life by arranging appropriate care. Prediction scores for short-term death in advanced oncology patients are present, but they are not validated for the ED and are mostly extensive assessment tools requiring patient information that is not always accessible in an emergency-setting [18].

To gain more knowledge on the course of events leading to ED visits at the end of life, the objectives of this study were to provide insight into characteristics of advanced cancer patients visiting the ED, their palliative care needs, and the actions undertaken during these ED visits.

## Methods

### Setting

This study was conducted at the Leiden University Medical Center (LUMC) in Leiden, the Netherlands. LUMC’s ED is open 24 h a day, 7 days a week. On average, 80 patients are evaluated every day for various reasons, including non-oncological problems. Since 2011, a palliative care consultation team (PCCT) is available in the LUMC for consultation of palliative patients.

### Patients

Adult patients who visited the ED between May 2011 and June 2014 were included who were in the palliative phase of cancer at the moment of the ED visit and died within 3 months thereafter. Patients were in the palliative phase if curation was not possible or if anti-cancer treatment was not directed at curation. The time period of 3 months represents the group of patients in urgent need of appropriate palliative care and appropriate end-of-life choices. Also, for the Dutch medical insurance system, this time period depicts the possibility of full reimbursement of necessary palliative home care or transfer to a hospice. Patients diagnosed with a haematological malignancy were excluded. Only data of the last ED visit before death were included.

### Data collection

Characteristics of the patients, referrals, and the ED visit and follow-up data were retrospectively collected from the electronic patients records (EPRs). The palliative disease phase was assigned by the researcher based on the disease trajectories described by Lynn and Adamson, in which three palliative phase can be discerned corresponding to the disease status: disease-modifying phase, in which anti-cancer treatment is given aimed at life prolongation or symptom management; symptom management phase, in which treatment is directed to symptom relief; or terminal phase [19]. EPRs were searched for notes reporting contact with general practitioners (GPs); for PCCT-consultations 3 months before the ED visit; and for proactive symptom management plans in files or letters until 6 weeks before the ED visit. Performance was scored using the Eastern Cooperative Oncology Group (ECOG) scale and was documented by the admitting hospital physician, or estimated by the researcher based on the patient’s physical functioning documented in the EPR [20]. Limitations on life-sustaining treatments included do-not-resuscitate orders, ‘no ventilation’-orders and ‘no intensive care unit (ICU) admission’-orders. The time of arrival at the hospital was defined as within office hours for visits from Monday to Friday between 8 a.m. and 6 p.m. The main symptom was defined as the symptom that led to the ED-referral as described in the EPR by the attending physician. New symptoms were defined as main symptoms not mentioned in the EPR 3 months before the ED visit. Acute symptoms were main symptoms with an onset within 24 h before the ED visit. The clinical diagnosis was defined as the conclusion of the attending ED physician.

### Statistics

Characteristics of patients, referral, and ED visit were analysed using descriptive statistics. Kaplan-Meier’s method was employed to estimate survival since the ED visit. The following factors associated with death were derived from literature search and clinical experience: primary lung tumour, ED-admissions for a new and acute problem, limitations on life-sustaining treatments before the ED visit, main symptom at the ED of neurologic deterioration, main symptom at the ED of dyspnoea, clinical diagnosis of bleeding, clinical diagnosis of cachexia, clinical diagnoses of hypercalcemia, and clinical diagnosis of jaundice. These factors were used in univariable and multivariable analyses by using a Cox proportional hazards regression. Predictors with a *p* value of < 0.10 in univariable analysis were entered in multivariable analysis. Differences with a *p* value < 0.05 were considered statistically significant. All analyses were conducted with SPSS 23.0 software.

## Results

### Patient characteristics

Four hundred twenty patients were included, median age was 63 years, and 229 (54.5%) patients were male (Table 1). Tumours located in the digestive tract occurred most frequently (27.6%). Anti-cancer treatment was provided to 73.6% of the patients in the 3 months before the ED visit. Most patients (62.6%) were in the disease-modifying palliative phase, with average time from diagnosis of the palliative phase to ED visit of 6.2 months (range 0–13.7 months). Most patients (92.6%) lived at home or in a residential home before the ED visit. Home care was arranged for 21.9% of the patients, of whom 10.5% received home care at least once a day. An informal caregiver was available for 87.1% of the patients. The PCCT was consulted for 26 patients (6.2%) in the last 3 months before the visit to the ED. Proactive symptom management plans were documented for 12.1% of the patients 6 weeks before the ED visit. Limitations on life-sustaining treatments had been discussed in 37.6% of the patients, and limitations had been documented in 33.8%.

**Table 1:** Four hundred twenty patients with advanced oncology visiting the emergency department

Patient characteristics	*N*	(% of 420)
Male	229	(54.5)
Age in years, median (range)	63	(22–92)
Primary tumour site		
Digestive tract	116	(27.6)
Lung	67	(16.0)
Gynecologic	47	(11.2)
Urologic	45	(10.7)
Breast	37	(8.8)
Head and neck	32	(7.6)
Other^a^		
Time since palliative diagnosis		
< 3 months	143	(34.0)
3 months–1 year	144	(34.3)
1 year–4 years	98	(23.3)
> 4 years	30	(7.1)
Palliative disease phase		
Disease-modifying	263	(62.6)
Symptom management	157	(37.4)
Treatment for primary tumour in the last 3 months		
Chemotherapy	168	(40.0)
Hormonal therapy	28	(6.7)
Targeted or immunotherapy	75	(17.9)
Radiotherapy	104	(24.8)
Surgery	31	(7.4)
Other^b^	7	(1.7)
None	111	(26.4)
Limitations on life-sustaining treatments		
Not discussed	262	(62.4)
Discussed, no limitations documented	16	(3.8)
Discussed, limitations documented^c^	142	(33.8)
Current housing situation^d^		
At home or residential home	389	(92.6)
Nursing home	12	(2.9)
Hospice	5	(1.2)
Home care		
No	225	(53.6)
Yes, unknown frequency	39	(9.3)
< 1×/day	9	(2.1)
≥1x/day	43	(10.5)
Informal caregiver available according to EPR	366	(87.1)
PCCT consulted during the last 3 months	26	(6.2)
Proactive symptom management plans		
In EPR, 6 weeks before the ED visit	51	(12.1)
In a letter to the GP, 6 weeks before the ED visit	30	(7.1)
Discussion with patient mentioned in EPR 6 weeks before the ED visit	46	(11.0)

*PCCT* palliative care consultation team, *EPR* electronic patient record, *ED* emergency department, *GP* general practitioner

^a^Other: other most common primary tumour sites were unknown primaries; skin tumours; sarcomas; and nasal cavity and middle ear

^b^Other: nuclear therapy (1%), hemo- or peritoneal dialysis (0.2%), organ transplantation (0.2%), and stem cell transplantation (0.2%)

^c^Documented limitations were:?? *n* (?%): no resuscitation: 62 (14.8%); no resuscitation, no ventilation: 11 (2.6%); no resuscitation, no ventilation, no admission to the intensive care unit: 68 (16.2%); refrain from any intervention: 1 (0.2%)

^d^Current living situation was not known for 14 patients (3.3%)

### Referral characteristics

Patients or their caregivers took the initiative to visit the ED in 34.0% for a median of 2.0 symptoms (Table 2). ED visits occurred outside office hours in 51.9%. The main symptom was new in 52.1% and acute in 36.9% of the patients and both new and acute in 29.3%. Most frequently reported main symptoms or signs were dyspnoea (21.0%), pain (18.6%), and ascites (11.9%). A total of 62.8% had an ECOG performance score of 3–4 (known in 196 of 420 patients).

**Table 2:** Referral of patients with advanced oncology to the emergency department

Referral characteristics	*N*	(% of 420)
Referrer		
GP or nursing home physician	150	(35.7)
GP out-of-office service	21	(5.0)
Medical specialist	100	(23.8)
Patient or informal caregiver	143	(34.0)
Referral outside office hours	218	(51.9)
Referral for		
a new problem^a^	219	(52.1)
an acute problem^b^	155	(36.9)
a new and acute problem	123	(29.3)
Number of symptoms, median (range)	2.0	(0–7)
Main symptom or sign for referral		
Dyspnoea	88	(21.0)
Pain	78	(18.6)
Ascites	50	(11.9)
Nausea or vomiting	39	(9.3)
Fever	38	(9.0)
Neurologic deterioration^c^	33	(7.9)
Bleeding	20	(4.8)
Weakness or loss of strength	19	(4.5)
Obstipation or diarrhoea	16	(3.8)
Difficulty swallowing or passage problems	9	(2.1)
Oedema	8	(1.9)
Seizure	8	(1.9)
Fatigue	8	(1.9)
WHO performance score		
0	4	(1.0)
1	26	(6.2)
2	43	(10.2)
3	89	(21.2)
4	34	(8.1)
Unknown	224	(53.3)

*ED* emergency department, *GP* general practitioner, *WHO* World Health Organization

^a^New problem: not reported in the patient records in the last 3 months

^b^Acute problem: originated within the last 24 h

^c^Neurologic deterioration: confusion, drowsiness, decreased consciousness

### Visit characteristics

At the ED, imaging and blood tests were performed in 63.3% and 83.3% of the patients, respectively (Table 3). Most frequently reported diagnoses by the attending physician were infection or fever (20.5%), bronchopulmonary insufficiency (12.9%), and renal insufficiency or hydronephrosis (11.2%). Patients spent a median time at the ED equal to 3:29 h (range 00:12–18:01). During or after the ED visit, limitations on life-sustaining treatments were discussed with 73.1% of the patients and 70.7% had limitations documented in the EPR. After the ED visit, 76.0% of the patients were hospitalized. Patients’ median survival from the ED visit was 18 days; 104 patients (24.8%) died within 1 week. Of the 104 patients who died within 1 week, 74 patients (71.2%) died in the hospital and death within 1 week was associated to in-hospital death (*p* < 0.0001, HR 8.49). In total, 39.3% of the patients died at home, 29.5% in a hospital (i.e. in the clinic, intensive care unit or another hospital) and 11.0% died in a hospice. In-hospital death occurred less frequently in patients with a proactive symptom management plan sent to their GP compared to patients without (26.9% and 38.5%, respectively, *p* = 0.03). In-hospital death was not related to limitations on life-sustaining treatments, the referrer or the number of previous admissions.

**Table 3:** Characteristics of ED visit and follow-up

Visit- and follow-up characteristics	*N*	(% of 420)
Diagnostic imaging	266	(63.3)
Laboratory tests performed	350	(83.3)
Clinical diagnosis		
Infection or fever	86	(20.5)
Bronchopulmonary insufficiency	54	(12.9)
Renal insufficiency or hydronephrosis	47	(11.2)
Cachexia	40	(9.5)
Ascites	34	(8.1)
Pleural effusion	31	(7.4)
Bleeding	30	(7.1)
Jaundice	23	(5.5)
Hypercalcemia	20	(4.8)
Ileus or passage disturbances	18	(4.3)
Neuropathy or plexopathy	17	(4.0)
Seizure	13	(3.1)
Urine retention	13	(3.1)
Fracture	10	(2.4)
Coma	8	(1.9)
Pulmonary embolism	8	(1.9)
Deep venous thrombosis	7	(1.7)
Delirium	6	(1.4)
Spinal cord compression	5	(1.2)
Any treatment initiated at ED	230	(54.8)
Time spent at ED, median (range)	03:29	(00:12–18:01)
Limitations on life-sustaining treatments^a^		
Discussed, none documented	10	(2.4)
Discussed and documented	297	(70.7)
Not discussed	113	(26.9)
Hospitalization after ED visit	319	(76.0)
Survival after ED visit in days, median (95% CI)	18	(15–21)
Death within 7 days after ED visit	104	(24.8)
Death within 14 days after ED visit	170	(40.5)
Death within 30 days after ED visit	274	(65.2)
Death within 60 days after ED visit	370	(88.1)
Place of death		
Hospital^b^	124	(29.5)
Home or residential home	165	(39.3)
Hospice	46	(11.0)
Nursing home	4	(1.0)
Unknown	81	(19.3)

*ED* emergency department, *h* hours, *mins* minutes, *IQ range* interquartile range, *ICU* intensive care unit

^a^During visit/after discharge

^b^One patient died at the ED (0.2%), 113 at a hospital ward (26.9%), and 10 at the ICU (2.4%)

### Factors associated with approaching death

Independent risk factors for early death were primary lung tumour (HR 1.69, 95% CI 1.29–2.21, *p* < 0.0001), referral for neurological deterioration (HR 2.01, 95% CI 1.38–2.92, *p* < 0.0001) or dyspnoea (HR 1.57, 95% CI 1.23–2.00) and hypercalcemia (HR 1.92, 95% CI 1.21–3.03, *p* = 0.005) or jaundice (HR 2.11, 95% CI 1.37–3.26, *p* = 0.001) (Table 4).

**Table 4:** Risk factors for death after ED visit

Predictors	Univariable analysis			Multivariable analysis		
	HR	95% CI	*p* value	HR	95% CI	*p* value
Primary lung tumour	1.67	1.28–2.18	< 0.0001	1.69	1.29–2.21	< 0.0001
ED-admission for new and acute problem	0.98	0.79–1.20	0.81			
Limitations on LSTs before ED visit	1.26	1.02–1.54	0.029			NS
Main symptom at the ED						
Neurologic deterioration	1.85	1.29–2.66	0.001	2.01	1.38–2.92	< 0.0001
Dyspnoea	1.48	1.17–1.88	0.001	1.57	1.23–2.00	< 0.0001
Clinical diagnosis						
Bleeding	1.37	0.95–1.99	0.096			NS
Cachexia	1.43	1.03–1.98	0.034			NS
Hypercalcemia	1.80	1.14–2.83	0.011	1.92	1.21–3.03	0.005
Jaundice	2.21	1.44–3.39	< 0.0001	2.11	1.37–3.26	0.001

*HR* hazard ratio, *CI* confidence interval, *LSTs* life-sustaining treatments, *ED* emergency department

## Discussion

This study provides a detailed description of patients with advanced cancer who visited the emergency department (ED) during the last 3 months of their lives and of the actions undertaken during these ED visits. In most patients, care seemed to focus on disease modification; many patients still received anticancer treatments, and few had proactive symptom management plans in case of progressive symptoms or limitations on life-sustaining treatments documented in their patient records. The ED visit triggered revision of limitations of life-sustaining treatments in the majority of patients. Following their ED visit, 76.0% was hospitalized in poor clinical condition and 29.5% died in the hospital; of those who died within 7 days, 71.2% died in-hospital. Factors associated with approaching death were found to aid identifying those patients with urgent palliative care needs at ED entry, in order to make appropriate decisions concerning their treatment and care trajectories.

ED staff, patients and their caregivers consider the ED setting an uncomfortable situation for patients at risk of approaching death [13]. Besides the hectic and noisy environment of the ED, there is little space for family members to stay with their sick relatives and to conduct end-of-life discussions. Palliative patients often have a lower priority than patients with acute life-threatening illnesses and therefore spend a lot of time waiting at the ED [15]. The overwhelming environment of the ED and uncertainty about the situation increases psychological distress and anxiety in patients and their caregivers [21]. For ED physicians, an important reason that makes it difficult to provide optimal care to palliative patients is that they have no long-lasting relationships [13, 22]. Moreover, they are not trained to provide adequate symptom management for and to discuss end-of-life decisions [13, 21, 22]. Notwithstanding, ED physicians are willing to provide palliative care and indicated that in order to enhance a ‘good death’, attention should be directed to the care needs and wishes of patients in the palliative phase visiting the ED [23, 24]. In our study, patients were exposed to many diagnostic tests (83% underwent blood tests, 63% diagnostic imaging) and stayed at the ED for 3.5 h on average, which was followed by hospitalization in over 75%. Since most patients prefer to spend the end of their life at home, these outcomes are undesirable [3].

Few patients in our study had limitations on life-sustaining treatments documented, suggesting that palliative care needs and approaching death had not yet been discussed. Patients and caregivers who are unprepared for or unaware of the problems and symptoms that may occur at the end of life are more likely to visit the ED at the end of life [15, 25], especially during out-of-office hours [8, 13, 26]. This is supported by our results: 34% of the patients referred themselves to the ED and 52% of the ED visits occurred out-of-office hours. Several studies reported that the majority of the ED visits are undesirable and avoidable, especially those by patients with a very short survival [5, 26, 27]. End-of-life discussions have shown to have the potential to prevent ED visits in the last month of life in patients with ovarian cancer [28] and stage IV lung and colorectal cancer [29]. Community-based palliative care effectively reduced the number of ED visits in the last phase of life in advanced cancer patients [30] and in the general patient population [31, 32]. Furthermore, meta-analysis of numerous randomized clinical trials proved that integration of palliative care early in the disease trajectory improves health-related quality of life and symptom intensity in patients with advanced cancer [33]. Advance care planning and out-patient symptom management may help patients and their caregivers to prepare for the end-of-life trajectory and to avoid unnecessary ED visits by supporting coping with deteriorating health [21, 34]. Although palliative care is often perceived as end-of-life care, palliative care can be provided concurrently with standard care [35]. Hence, timely initiation of palliative care is possible and helps to avoid unnecessary ED visits and can improve quality of life in the end-of-life phase.

Although early palliative care can avoid part of the ED visits at the end of life, there will still be patients visiting the ED for symptoms that are distressing and unmanageable at home. Additionally, patients may visit the ED when community palliative care services are not available, e.g., outside office hours [15, 21]. ED visits can be an opportunity to recognize high symptom burden and acute deterioration, which should trigger initiation of appropriate palliative care. This is also known as ED-initiated palliative care [36, 37]. Grudzen et al. conducted a randomized clinical trial in 2016 on palliative care consultations initiated at the ED in patients with advanced cancer and found that it significantly improved their quality of life [37]. Examples of ED-initiated palliative care are, among others, consultations by a specialized in-hospital team, community-based care by a homecare team or hospice team, telephone-based interventions, or admissions to a hospice or a palliative care unit [33]. Our finding that physicians documented more limitations on LSTs after the ED visit might indicate that they were well aware of changes in disease trajectories, creating an opportunity for effective ED-initiated palliative care. To facilitate cooperation with palliative care services, both at home and in the hospital, it is recommended to have a checklist with standardized criteria [38] for referral with contact details of the palliative care services easily available at the ED. An international consensus panel of 60 experts on palliative cancer care formulated 11 criteria for referral to specialized palliative care: nine needs-based criteria (severe physical symptoms, severe emotional symptoms, request for hastened death, spiritual or existential crisis, need for assistance with decision-making or care planning, referral on patient’s request, delirium, brain or leptomeningeal metastases, spinal cord compression or cauda equine) and two time-based criteria (within 3 months of diagnosis of advanced cancer or incurable cancer for patients with a median survival of 1 year of less, diagnosis of advanced cancer with progressive disease despite second-line systemic therapy) [39]. The severity of symptoms can be measured by using the Edmonton Symptom Assessment Scale (ESAS), a patient-reported outcome measure for symptoms prevalent in the palliative phase which is manageable at the ED [40, 41]. Although the ESAS is not yet validated in the ED setting, a study by Barbera et al. shows that poor symptom burden scores were associated with higher usage of the ED, suggesting that patients visit the ED particularly with high palliative care needs which should be acted upon as soon as possible [42].

**Fig. 1:**
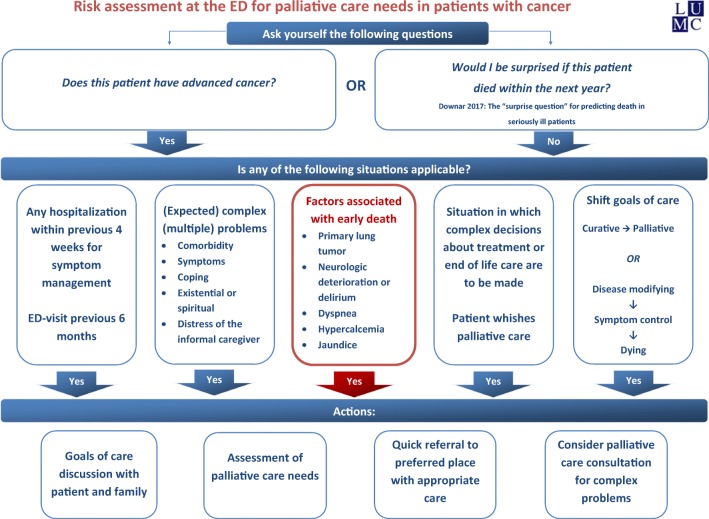
Risk assessment at the ED for palliative care needs in patients with cancer

To identify patients in whom palliative care should be initiated, survival prediction tools such as the Surprise Question, and prediction scores such as the Palliative Prognostic Score (PaP), Palliative Prognostic Index (PPI), Glascow Prognostic Score (GPS) and Prognosis in Palliative Care Study (PiPS) are described [18, 43]. However, these tools are not validated in patients with advanced cancer visiting the ED. To facilitate appropriate and ED-initiated palliative care, we constructed a flowchart to help ED staff identify advanced cancer patients with urgent palliative care needs (Fig. 1). In this flowchart, factors from the current study associated with approaching death, suggesting urgent palliative care needs, are depicted: primary lung tumour, dyspnoea, neurologic deterioration, jaundice and hypercalcemia. Other known triggers for palliative care needs that are easily assessable at the ED were added to the flowchart. In other studies in advanced cancer patients, dyspnoea and respiratory distress are reported as risk factors for approaching death, as are neurological deterioration and gastro-intestinal problems [9, 44, 45]. Hypercalcemia is probably predictive of death because it can be a marker for progressive disease in patients with bone metastases or paraneoplastic syndromes [46]. Cachexia was associated with approaching death in our univariable model, and delirium was included in the group with neurological deterioration. Although a decline in performance status is a strong predictor for death [47, 48], we could not find an association with death, probably because values were missing for many patients. If advanced cancer patients with urgent palliative care needs are identified at the ED, ED staff may choose to consult the hospital palliative care consultation team. Also, tools for unmet palliative needs screening are available, such as the ‘Screen for Palliative and End-of-life care needs in the Emergency Department (SPEED)’ tool [49] or the shorter 5-SPEED tool [50]. The SPEED is the only palliative care needs assessment tool that is validated for use at the ED; however, it is not yet validated in patients with advanced cancer.

This pragmatic study gives insight into the end-of-life trajectory of patients with advanced cancer who visit the ED. We are aware that the retrospective design of our study could have led to registration bias and unmeasured confounding. Selection bias was introduced by the choice to limit inclusion to cancer patients in the palliative phase of their disease who died within 3 months after the ED visit. We aimed to describe the population of advanced cancer patients who visited the ED at the end of their life, because especially in those patients, appropriate care should be initiated at the ED. Lastly, because the end-of-life trajectory, especially in the last 3 months, has not been subject to major changes, we consider our data collected from 2011 to 2014 still relevant to the present situation. Further research should be conducted to validate survival prediction tools and needs assessment tools for patients with advanced cancer visiting the ED and to evaluate implementation of models of ED-initiated palliative care.

## Conclusion

Advanced cancer patients received limited palliative care before visiting the ED in the last 3 months of their life. The ED visit often marked physical deterioration and triggered revision of limitations on life-sustaining treatments. Many patients were hospitalized and a substantial percentage died within 1 week and in-hospital. Timely recognition of patients at high risk of approaching death and awareness of the potential of ED-initiated palliative care among ED-staff can improve the end-of-life trajectory of these patients.

## References

[1] Fitzmaurice C, Akinyemiju TF, Al Lami FH, Alam T, Alizadeh-Navaei R, Allen C, Alsharif U, Alvis-Guzman N, Amini E, Anderson BO, Aremu O, Artaman A, Asgedom SW, Assadi R, Atey TM, Avila-Burgos L, Awasthi A, Ba Saleem HO, Barac A, Bennett JR, Bensenor IM, Bhakta N, Brenner H, Cahuana-Hurtado L, Castaneda-Orjuela CA, Catala-Lopez F, Choi JJ, Christopher DJ, Chung SC, Curado MP, Dandona L, Dandona R, das Neves J, Dey S, Dharmaratne SD, Doku DT, Driscoll TR, Dubey M, Ebrahimi H, Edessa D, El-Khatib Z, Endries AY, Fischer F, Force LM, Foreman KJ, Gebrehiwot SW, Gopalani SV, Grosso G, Gupta R, Gyawali B, Hamadeh RR, Hamidi S, Harvey J, Hassen HY, Hay RJ, Hay SI, Heibati B, Hiluf MK, Horita N, Hosgood HD, Ilesanmi OS, Innos K, Islami F, Jakovljevic MB, Johnson SC, Jonas JB, Kasaeian A, Kassa TD, Khader YS, Khan EA, Khan G, Khang YH, Khosravi MH, Khubchandani J, Kopec JA, Kumar GA, Kutz M, Lad DP, Lafranconi A, Lan Q, Legesse Y, Leigh J, Linn S, Lunevicius R, Majeed A, Malekzadeh R, Malta DC, Mantovani LG, McMahon BJ, Meier T, Melaku YA, Melku M, Memiah P, Mendoza W, Meretoja TJ, Mezgebe HB, Miller TR, Mohammed S, Mokdad AH, Moosazadeh M, Moraga P, Mousavi SM, Nangia V, Nguyen CT, Nong VM, Ogbo FA, Olagunju AT, Pa M, Park EK, Patel T, Pereira DM, Pishgar F, Postma MJ, Pourmalek F, Qorbani M, Rafay A, Rawaf S, Rawaf DL, Roshandel G, Safiri S, Salimzadeh H, Sanabria JR, Santric Milicevic MM, Sartorius B, Satpathy M, Sepanlou SG, Shackelford KA, Shaikh MA, Sharif-Alhoseini M, She J, Shin MJ, Shiue I, Shrime MG, Sinke AH, Sisay M, Sligar A, Sufiyan MB, Sykes BL, Tabares-Seisdedos R, Tessema GA, Topor-Madry R, Tran TT, Tran BX, Ukwaja KN, Vlassov VV, Vollset SE, Weiderpass E, Williams HC, Yimer NB, Yonemoto N, Younis MZ, Murray CJL, Naghavi M, Global Burden of Disease Cancer C (2018). Global, regional, and National Cancer Incidence, mortality, years of life lost, years lived with disability, and disability-adjusted life-years for 29 Cancer groups, 1990 to 2016: a systematic analysis for the global burden of disease study. JAMA Oncol.

[2] World Health Organization (2018) WHO Definition of Palliative Care. http://www.who.int/cancer/palliative/definition/en/. Accessed 5 Oct 2018

[3] Gomes B, Higginson IJ, Calanzani N, Cohen J, Deliens L, Daveson BA, Bechinger-English D, Bausewein C, Ferreira PL, Toscani F, Menaca A, Gysels M, Ceulemans L, Simon ST, Pasman HR, Albers G, Hall S, Murtagh FE, Haugen DF, Downing J, Koffman J, Pettenati F, Finetti S, Antunes B, Harding R, Prisma (2012). Preferences for place of death if faced with advanced cancer: a population survey in England, Flanders, Germany, Italy, the Netherlands, Portugal and Spain. Ann Oncol.

[4] Higginson IJ, Sen-Gupta GJ (2000). Place of care in advanced cancer: a qualitative systematic literature review of patient preferences. J Palliat Med.

[5] Barbera L, Taylor C, Dudgeon D (2010). Why do patients with cancer visit the emergency department near the end of life?. CMAJ.

[6] Earle CC, Neville BA, Landrum MB, Ayanian JZ, Block SD, Weeks JC (2004). Trends in the aggressiveness of cancer care near the end of life. J Clin Oncol.

[7] Qureshi D, Tanuseputro P, Perez R, Seow H (2018). Place of Care trajectories in the last two Weeks of life: a population-based cohort study of Ontario decedents. J Palliat Med.

[8] Mayer DK, Travers D, Wyss A, Leak A, Waller A (2011). Why do patients with cancer visit emergency departments? Results of a 2008 population study in North Carolina. J Clin Oncol.

[9] Geraci JM, Tsang W, Valdres RV, Escalante CP (2006). Progressive disease in patients with cancer presenting to an emergency room with acute symptoms predicts short-term mortality. Support Care Cancer.

[10] Mercadante S, Porzio G, Valle A, Aielli F, Costanzo V, Adile C, Spedale V, Casuccio A, Home Care Italy G (2012). Emergencies in patients with advanced cancer followed at home. J Pain Symptom Manag.

[11] Mercadante S, Masedu F, Valenti M, Mercadante A, Aielli F (2016). The characteristics of advanced cancer patients followed at home, but admitted to the hospital for the last days of life. Intern Emerg Med.

[12] Henson LA, Higginson IJ, Daveson BA, Ellis-Smith C, Koffman J, Morgan M, Gao W, BuildCare (2016). I'll be in a safe place': a qualitative study of the decisions taken by people with advanced cancer to seek emergency department care. BMJ Open.

[13] Cooper E, Hutchinson A, Sheikh Z, Taylor P, Townend W, Johnson MJ (2018). Palliative care in the emergency department: a systematic literature qualitative review and thematic synthesis. Palliat Med.

[14] Jelinek GA, Marck CH, Weiland TJ, Philip J, Boughey M, Weil J, Lane H (2013). Caught in the middle: tensions around the emergency department care of people with advanced cancer. Emerg Med Australas.

[15] Smith AK, Fisher J, Schonberg MA, Pallin DJ, Block SD, Forrow L, Phillips RS, McCarthy EP (2009). Am I doing the right thing? Provider perspectives on improving palliative care in the emergency department. Ann Emerg Med.

[16] Marck CH, Weil J, Lane H, Weiland TJ, Philip J, Boughey M, Jelinek GA (2014). Care of the dying cancer patient in the emergency department: findings from a national survey of Australian emergency department clinicians. Intern Med J.

[17] Lane H, Weil J, Jelinek GA, Boughey M, Marck CH, Weiland TJ, Haydon A, Philip J (2014). Ideal care and the realities of practice: interdisciplinary relationships in the management of advanced cancer patients in Australian emergency departments. Support Care Cancer.

[18] Hui D (2015). Prognostication of survival in patients with advanced Cancer: predicting the unpredictable?. Cancer Control.

[19] Lynn Joanne ADM (2003). Living Well at the End of Life. Adapting Health Care to Serious Chronic Illness in Old Age.

[20] Oken MM, Creech RH, Tormey DC, Horton J, Davis TE, McFadden ET, Carbone PP (1982). Toxicity and response criteria of the eastern cooperative oncology group. Am J Clin Oncol.

[21] Smith AK, Schonberg MA, Fisher J, Pallin DJ, Block SD, Forrow L, McCarthy EP (2010). Emergency department experiences of acutely symptomatic patients with terminal illness and their family caregivers. J Pain Symptom Manag.

[22] Stone SC, Mohanty S, Grudzen CR, Shoenberger J, Asch S, Kubricek K, Lorenz KA (2011). Emergency medicine physicians' perspectives of providing palliative care in an emergency department. J Palliat Med.

[23] Decker K, Lee S, Morphet J (2015). The experiences of emergency nurses in providing end-of-life care to patients in the emergency department. Australas Emerg Nurs J.

[24] Shearer FM, Rogers IR, Monterosso L, Ross-Adjie G, Rogers JR (2014). Understanding emergency department staff needs and perceptions in the provision of palliative care. Emerg Med Australas.

[25] Korte-Verhoef R (2014). Reasons and Avoidability of Hospitalisations at the End of Life. Perspectives of GPs, Nurses and Family Carers.

[26] Wallace EM, Cooney MC, Walsh J, Conroy M, Twomey F (2013). Why do palliative care patients present to the emergency department? Avoidable or unavoidable?. Am J Hosp Palliat Care.

[27] Delgado-Guay MO, Kim YJ, Shin SH, Chisholm G, Williams J, Allo J, Bruera E (2015). Avoidable and unavoidable visits to the emergency department among patients with advanced cancer receiving outpatient palliative care. J Pain Symptom Manag.

[28] Lopez-Acevedo M, Havrilesky LJ, Broadwater G, Kamal AH, Abernethy AP, Berchuck A, Alvarez Secord A, Tulsky JA, Valea F, Lee PS (2013). Timing of end-of-life care discussion with performance on end-of-life quality indicators in ovarian cancer. Gynecol Oncol.

[29] Mack JW, Cronin A, Keating NL, Taback N, Huskamp HA, Malin JL, Earle CC, Weeks JC (2012). Associations between end-of-life discussion characteristics and care received near death: a prospective cohort study. J Clin Oncol.

[30] Henson LA, Gao W, Higginson IJ, Smith M, Davies JM, Ellis-Smith C, Daveson BA (2015). Emergency department attendance by patients with cancer in their last month of life: a systematic review and meta-analysis. J Clin Oncol.

[31] Spilsbury K, Rosenwax L, Arendts G, Semmens JB (2017). The Association of Community-Based Palliative Care with Reduced Emergency Department Visits in the last year of life varies by patient factors. Ann Emerg Med.

[32] Spilsbury K, Rosenwax L, Arendts G, Semmens JB (2017). The impact of community-based palliative care on acute hospital use in the last year of life is modified by time to death, age and underlying cause of death. A population-based retrospective cohort study. PLoS One.

[33] Haun MW, Estel S, Rucker G, Friederich HC, Villalobos M, Thomas M, Hartmann M (2017) Early palliative care for adults with advanced cancer. Cochrane Database Syst Rev (6):CD011129. 10.1002/14651858.CD011129.pub2 10.1002/14651858.CD011129.pub2 PMC6481832 28603881

[34] Wright CM, Youens D, Moorin RE (2018). Earlier initiation of community-based palliative Care is associated with fewer unplanned hospitalizations and emergency department presentations in the final months of life: a population-based study among Cancer decedents. J Pain Symptom Manag.

[35] Vanbutsele G, Pardon K, Van Belle S, Surmont V, De Laat M, Colman R, Eecloo K, Cocquyt V, Geboes K, Deliens L (2018). Effect of early and systematic integration of palliative care in patients with advanced cancer: a randomised controlled trial. Lancet Oncol.

[36] Kistler EA, Sean Morrison R, Richardson LD, Ortiz JM, Grudzen CR (2015). Emergency department-triggered palliative care in advanced cancer: proof of concept. Acad Emerg Med.

[37] Grudzen CR, Richardson LD, Johnson PN, Hu M, Wang B, Ortiz JM, Kistler EA, Chen A, Morrison RS (2016). Emergency department-initiated palliative Care in Advanced Cancer: a randomized clinical trial. JAMA Oncol.

[38] Hui D, Hannon BL, Zimmermann C, Bruera E (2018). Improving patient and caregiver outcomes in oncology: team-based, timely, and targeted palliative care. CA Cancer J Clin.

[39] Hui D, Mori M, Watanabe SM, Caraceni A, Strasser F, Saarto T, Cherny N, Glare P, Kaasa S, Bruera E (2016). Referral criteria for outpatient specialty palliative cancer care: an international consensus. Lancet Oncol.

[40] Hui D, Bruera E (2017). The Edmonton symptom assessment system 25 years later: past, present, and future developments. J Pain Symptom Manag.

[41] Hoerger M, Greer JA, Jackson VA, Park ER, Pirl WF, El-Jawahri A, Gallagher ER, Hagan T, Jacobsen J, Perry LM, Temel JS (2018). Defining the elements of early palliative Care that are associated with patient-reported outcomes and the delivery of end-of-life Care. J Clin Oncol.

[42] Barbera L, Atzema C, Sutradhar R, Seow H, Howell D, Husain A, Sussman J, Earle C, Liu Y, Dudgeon D (2013). Do patient-reported symptoms predict emergency department visits in cancer patients? A population-based analysis. Ann Emerg Med.

[43] Downar J, Goldman R, Pinto R, Englesakis M, Adhikari NK (2017). The "surprise question" for predicting death in seriously ill patients: a systematic review and meta-analysis. CMAJ.

[44] Maltoni M, Caraceni A, Brunelli C, Broeckaert B, Christakis N, Eychmueller S, Glare P, Nabal M, Vigano A, Larkin P, De Conno F, Hanks G, Kaasa S, Steering Committee of the European Association for Palliative C (2005). Prognostic factors in advanced cancer patients: evidence-based clinical recommendations--a study by the steering Committee of the European Association for palliative Care. J Clin Oncol.

[45] Trajkovic-Vidakovic M, de Graeff A, Voest EE, Teunissen SC (2012). Symptoms tell it all: a systematic review of the value of symptom assessment to predict survival in advanced cancer patients. Crit Rev Oncol Hematol.

[46] de Graeff A KR (2009) Richtlijn Hypercalciemie. https://www.oncoline.nl/hypercalciemie. Accessed 16-08-2018

[47] Seow H, Barbera L, Sutradhar R, Howell D, Dudgeon D, Atzema C, Liu Y, Husain A, Sussman J, Earle C (2011). Trajectory of performance status and symptom scores for patients with cancer during the last six months of life. J Clin Oncol.

[48] Evans C, McCarthy M (1985). Prognostic uncertainty in terminal care: can the Karnofsky index help?. Lancet.

[49] George N, Barrett N, McPeake L, Goett R, Anderson K, Baird J (2015). Content validation of a novel screening tool to identify emergency department patients with significant palliative Care needs. Acad Emerg Med.

[50] Reuter Quentin, Marshall Alison, Zaidi Hashim, Sista Priyanka, Powell Emilie S., McCarthy Danielle M., Dresden Scott M. (2019). Emergency Department-Based Palliative Interventions: A Novel Approach to Palliative Care in the Emergency Department. Journal of Palliative Medicine.

